# The Transplant Experience for Undocumented Immigrant Patients Formerly Receiving Emergency Dialysis and Caregivers

**DOI:** 10.1001/jamanetworkopen.2023.54602

**Published:** 2024-02-29

**Authors:** Katherine Rizzolo, Nathan Rockey, Claudia Camacho, Colin Gardner, Sixto Giusti, Lilia Cervantes

**Affiliations:** 1 Section of Nephrology, Boston University Chobanian and Avedisian School of Medicine and Boston Medical Center, Boston, Massachusetts; 2 Department of General Internal Medicine, University of Colorado Anschutz Medical Campus, Aurora; 3 University of Colorado Medical School, University of Colorado Anschutz Medical Campus, Aurora; 4 Department of Transplant, University of Colorado Anschutz Medical Campus, Aurora

## Abstract

**Importance:**

Individuals of undocumented immigration status with kidney failure face barriers to receiving transplants due to lack of health insurance despite no regulatory barriers. Little is known about the perspectives on kidney transplant among individuals with undocumented immigration status with kidney failure who relied on emergency hemodialysis.

**Objective:**

To examine the overall experiences of transplant among transplant recipients of undocumented immigration status who previously relied on emergency hemodialysis and their family caregivers.

**Design, Setting, and Participants:**

In this qualitative study, semistructured 1-to-1 interviews were conducted with transplant recipients who had previously received emergency hemodialysis and transitioned to scheduled dialysis and their primary caregivers living in Denver, Colorado, between May 1, 2022, and March 31, 2023, in English or Spanish.

**Main Outcomes and Measures:**

Themes and subthemes regarding the experience of transplant as an undocumented immigrant previously receiving emergency hemodialysis were identified. Interview transcripts were translated, deidentified, and then analyzed using thematic analysis.

**Results:**

A total of 25 participants including 15 transplant recipients (5 [33.3%] female and 10 [66.7%] male; mean [SD] age, 49.5 [9.8] years) and 10 caregivers (7 [70.0%] female and 3 [30.0%] male; mean [SD] age, 44.5 [22.3] years) participated. Six themes were reported: limited kidney replacement therapy education while receiving emergency hemodialysis (lack of awareness of kidney disease and treatment options and discriminatory kidney replacement therapy education due to immigration status), hope for transplant once receiving scheduled dialysis (prospect of transplant through scheduled dialysis, family and quality of life as transplant motivators), transplant education and health insurance after transition to scheduled dialysis (inadequate transplant education in dialysis clinic, peer-to-peer transplant education, and peer-to-peer communication regarding availability of private health insurance), uncertainty during transplant evaluation (difficulty navigating the evaluation and wait-listing process, lack of communication regarding timeline, and concern for family limiting living donation), posttransplant improvements (ability to work after transplant is critically important given immigration status, autonomy with transplant improves mental health, and vigilance in maintaining transplant), and transplant facilitators (self-advocacy, spirituality and optimism, and peer support).

**Conclusions and Relevance:**

This qualitative study of transplant recipients of undocumented immigration status and their caregivers found that individuals formerly receiving emergency dialysis are excluded from education and access to transplant, and peer support throughout the transplant process helped with education and motivation to pursue transplant. These findings may be used to implement improvements in access to support and education for patients of undocumented immigration status with kidney failure, especially in areas where scheduled dialysis is not available.

## Key Points

**Question:**

What is the transplant experience for individuals of undocumented immigration status with kidney failure who previously received emergency hemodialysis and for their caregivers?

**Findings:**

In this qualitative study of 15 transplant recipients of undocumented immigration status and 10 caregivers, participants received minimal education regarding transplant and insurance. Moving to scheduled dialysis facilitated motivation for transplant and peer-to-peer education.

**Meaning:**

Addressing education gaps and improving access to scheduled dialysis and transplant for undocumented individuals with kidney failure may help improve the transplant experience for individuals of undocumented immigration status.

## Teaser

This qualitative study examines the transplant experience among kidney transplant recipients of undocumented immigration status who previously relied on emergency hemodialysis and their family caregivers.

## Introduction

Individuals of undocumented immigration status in the US are excluded from federal insurance options, such as Medicare and federal insurance provisions under the Patient Protection and Affordable Care Act, leading to especially deleterious effects for those with kidney failure. Access to dialysis and transplant varies widely by state. Little is known about the experience of transplant recipients of undocumented immigration status in areas without statewide access to transplants.

Citizenship does not preclude eligibility for transplant in the US. However, lack of affordable insurance coverage is a major structural barrier to receiving transplants because it is a prerequisite for transplantation at most transplant centers.^1^ As of 2022, there are 5 states that offer statewide transplant health care coverage to individuals of undocumented immigration status with kidney failure through inclusion in Medicaid, statewide funding designated for transplant under Emergency Medicaid, private statewide charity funding, or high-risk insurance pools.^2^ In states without statewide provisions for transplant care, insurance options for people of undocumented immigration status are limited to insurance purchased off the Marketplace exchange or receipt of nationally available charity funding, which pays premiums on private insurance.^3,4^ However, these charity funds pay for transplant care for the first calendar year of transplant, restricting access to transplant to those who are able to secure funding to pay for posttransplant care after this first year.

Statewide affordable mechanisms for providing scheduled dialysis care are used in 20 US states and Washington, DC, via provisions under Emergency Medicaid, inclusion in Medicaid, or access to high-risk insurance pools.^2^ In other states, many individuals of undocumented immigration status remain without provisions for dialysis care and are forced to receive dialysis care through the emergency department, which is associated with a 14-fold higher risk of death,^5^ a higher symptom burden,^6^ and caregivers and clinicians experiencing emotional distress.^7^ In Colorado, before 2019, individuals of undocumented immigration status were able to access only emergency dialysis but were able to switch to scheduled dialysis after a 2019 change in the provision of coverage under Emergency Medicaid that provided coverage of services related to kidney failure but did not cover transplant.

Given the multiple unique barriers in access to transplants facing individuals of undocumented immigration status, exploring what factors and resources enabled success in transplant for this vulnerable population can help improve care for this population with similar access barriers in other states. The objective of this study was to examine the experience of transplant among transplant recipients of undocumented immigration status who previously relied on emergency hemodialysis and their family caregivers to outline the factors critical to transplant success.

## Methods

### Study Design

Semistructured 1-on-1 interviews were conducted between May 1, 2022, and March 31, 2023, in English or Spanish. Participants provided verbal informed consent and received $60 compensation. This qualitative study followed the Consolidated Criteria for Reporting Qualitative Research (COREQ) reporting guideline.^8^ The Colorado Multi-Institutional Review Board approved the study.

### Setting and Participants

Eligible participants were adult (aged ≥18 years) transplant recipients who formerly received emergency dialysis before switching to scheduled dialysis and their (patient-identified) primary caregivers living in Denver, Colorado. Participants were identified from the University of Colorado Transplant Center patient database and recruited via convenience sampling. The invitation to participate occurred by telephone; the semistructured interview and demographic questionnaire occurred via telephone or in person.

### Interview Guide

The interview guide (eTable in Supplement 1) was informed by literature review,^5,6,7,9^ which was used to identify sensitizing concepts for the interview.^10^ Questions were iteratively developed through the interview process and explored the experience receiving emergency and scheduled dialysis, experiences with transplant education and evaluation, motivations for transplant, and life after transplant.

### Data Collection

All interviews were conducted in Spanish by bilingual members of the study team (N.R., C.G., and C.C.). The study team consisted of an internal medicine physician (N.R.) and a medical student (C.G.); both were trained in qualitative interviewing by a nephrologist with prior qualitative research experience (K.R.). Neither had previous clinical interactions with participants. One member of the study team (C.C.) is a Latina patient navigator well trained in qualitative interviewing who had previous clinical interactions with participants. Interviews were audio-recorded, transcribed verbatim, professionally translated to English, and deidentified. Interviews continued until thematic saturation (ie, when further observation and analysis do not reveal new concepts).^11^

### Analysis

Interview transcripts were contemporaneously analyzed by 2 members (K.R. and N.R.) using atlas.ti, version 9.22 (ATLAS.ti Scientific Software Development GmbH) between August 1, 2022, and March 31, 2023. Coding and analysis were performed to inductively identify concepts utilizing thematic analysis.^11,12^ These concepts were grouped into initial themes and subthemes, and a theoretical framework was developed through process of analysis and comparison of concepts using principles of grounded theory.^10,11,13^ Consensus on themes and subthemes was reached after review of the thematic analysis to ensure that the findings reflected the full range and depth of the data. Member-checking (returning the data to participants for accuracy) was not conducted.^14^

## Results

A total of 25 participants, including 15 transplant recipients (5 [33.3%] female and 10 [66.7%] male; mean [SD] age, 49.5 [9.8] years) and 10 caregivers (7 [70.0%] female and 3 [30.0%] male; mean [SD] age, 44.5 [22.3] years), participated. Fifteen of 19 potential transplant recipient participants were interviewed (Table 1). Reasons for nonparticipation included lack of interest and no time (n = 2) and verifying they did not receive emergency dialysis (n = 2). Of the transplant recipients, 14 (93.3%) were from Mexico and 1 (6.7%) was from El Salvador. The transplant recipients had spent a mean (SD) of 21.9 (9.8) years in the US. Thirteen (86.7%) received a deceased donor transplant. Transplant recipients received care at a total of 4 emergency departments and 8 outpatient dialysis centers.

**Table 1.** Characteristics of the Transplant Recipients and Caregivers^a^

Characteristic	Findings
**Transplant recipients (n = 15)**	
Sex	
Female	5 (33.3)
Male	10 (66.7)
Age, mean (SD), y	49.5 (9.8)
Country of origin	
Mexico	14 (93.3)
El Salvador	1 (6.7)
Married or with a partner	6 (40)
English proficiency	
None or poor	9 (60.0)
Basic	4 (26.7)
Moderate or fluent	2 (13.3)
Time in US, mean (SD), y	21.9 (9.8)
Full-time employment after transplant	5 (33.3)
Less than high school education	7 (46.7)
Time receiving dialysis, mean (SD), y	5.7 (2.1)
Receipt of predialysis care	3 (20)
Deceased donor transplant	13 (86.7)
History of diabetes	4 (26.7)
**Caregivers (n = 10)**	
Sex	
Female	7 (70.0)
Male	3 (30.0)
Age, mean (SD), y	44.5 (22.3)
English proficiency	
None or poor	0
Basic	7 (70.0)
Moderate or fluent	3 (30.0)
Relationship to recipient	
Spouse	5 (50.0)
Sibling	2 (20.0)
Parent	1 (10.0)
Child	2 (20.0)
Recipient received a deceased donor transplant	8 (80.0)
No. of dependents, mean (SD)	1.3 (1.1)

^a^ Data are presented as number (percentage) of recipients or caregivers unless otherwise indicated.

Ten of 12 caregivers were interviewed. Reasons for nonparticipation included lack of interest and time (n = 2). Caregivers included spouses (n = 5), siblings (n = 2), a parent of a recipient (n = 1), and children of recipients (n = 2). The mean (SD) interview duration for transplant recipients and caregivers was 75 (22.2) minutes. We identified 6 themes (Table 2), and a thematic schema was developed to illustrate relationships among themes (eFigure in Supplement 1).

**Table 2.** Themes and Subthemes With Illustrative Quotations^a^

Themes and subthemes	Quotations
Limited kidney replacement therapy education while receiving emergency hemodialysis	
Lack of awareness of kidney disease and treatment options	“I still remember the day we were visiting a family when I received the call that it was time to start dialysis. And I remember that honestly, I didn’t know what I was getting myself into.” (Patient 11)
	“I never thought that dialysis was cleaning blood, that they place a catheter, and all that. I just thought that it was something where they operated you and you were out of there.” (Patient 12)
	“I thought that my kidneys were going to be cured with medication and the doctor said no, that’s my kidneys weren’t functioning anymore, that I needed dialysis because if I didn’t get dialysis my life was going to be over in like 3 or 4 days and then I was going to die. That’s what the doctor told me. I got scared, I wanted to run out of the hospital.” (Patient 5)
Discriminatory kidney replacement therapy education due to immigration status	“I started dialysis because it was an emergency. We are not allowed to have it otherwise. Because of the lack of documents, we didn’t have access to normal care like a person who has documents.” (Patient 6)
	“We were on dialysis every 8 days. This dialysis was given to us as emergency dialysis to save our lives. Nothing more. It was not like to survive or lead a more normal daily life depending on the disease. It was just to not lose our life.” (Patient 1)
	“When you arrive at a hospital without documents and health insurance, sadly they treat you as if your life is worthless.” (Patient 2)
	“They always scolded me that my potassium was really high and this and that and that it was dangerous. But I would tell them, ‘Well, how do I measure it if sometimes I come here doing well and they don’t want to treat me? I have to come really sick to be treated.’” (Patient 12)
Hope for transplant once receiving scheduled dialysis	
Prospect of transplant through scheduled dialysis	“When they started doing the dialysis 3 times a week, it changed my life. I felt better. When they gave me dialysis, I felt good, I ate and I was relieved, the next day I went to work well, so it did improve my life.” (Patient 3)
	“With that dialysis I could see she got her appetite back…I could see in her face the brilliance that you see in the face of a person who is a bit more normal.” (Caregiver 1)
	“Well, we were better and it gave us hope. It gave us hope because they told us that, thanks to that insurance, he might be able to have a transplant.” (Caregiver 8)
	“I think that it [the possibility of transplant] gives hope to the person on dialysis...and they will try. Because it wasn’t like this before.” (Caregiver 2)
Family and quality of life as transplant motivators	“For me, for my family. My daughters, and my wife. I knew they all struggled. I could tell they were suffering too.” (Patient 3)
	“I wanted to feel fine, and my children, and I didn’t want to be a burden to my siblings. Because they work, and I didn’t want to depend on them.” (Patient 15)
	“I wanted to be happy and feel like I did before and I wanted to work like I used to work before and if one day I want to go see my mom or my dad, my family.” (Patient 5)
Transplant education and health insurance after transition to scheduled dialysis	
Inadequate transplant education in dialysis clinic	“They never talked to me about it, one day I asked a nurse, but since I was speaking in Spanish and I don’t speak English to have a conversation, she said, ‘Another day we’ll talk.’” (Patient 3)
	“They didn’t mention much about the financial barriers and I didn’t ask for help either.” (Patient 8)
	“I have friends who…are not told, ‘We can put the translator there for you so you can do it.’ I have seen how they last for months because the social worker is never there. Or because the one who receives the call never tells her. There is a lack of humility there. Lack of interest in helping.” (Patient 2)
Peer-to-peer transplant education	“When I was getting dialysis, I was given the information about the transplant...[from] a friend who was going to dialysis at the clinic.” (Patient 3)
	“While I was on dialysis, I met a Mexican friend and he told me, ‘I am going to a transplant place in Denver. I am going to have an appointment and I am going to talk about you and see what they say because you have insurance, to see if they will put you on the waiting list for a transplant.’” (Patient 3)
	“We were in contact with each other…that was the way we started to open that door to start this whole transplant procedure.” (Patient 1)
Peer-to-peer communication regarding availability of private health insurance	“He met someone who got a transplant and the guy told him to look for an insurance.…They told me that tomorrow there will be talks about getting insurance to see if there is an opportunity for me to get a transplant.” (Caregiver 2)
	“[A friend] said, ‘Look, now that I went through this process, I’m going to tell you how to do it.’ So that’s when he sent me to buy the insurance.” (Patient 12)
	“The insurance we got was available for some years and we didn’t have the information.…So, more than anything is the information and you have to know where to go, because if you wait for someone to tell you that, it’ll be too difficult.” (Patient 4)
Uncertainty during transplant evaluation	
Difficulty navigating the evaluation and wait-listing process	“You need to be very good and your levels need to be a certain place for a long time…you have no reason to give up hope but at the same time you have to have all those levels at a good level.” (Caregiver 2)
	“One time, when they did the test there was a little problem there. I don’t know what happened for her, but she started to bleed a lot…I thought it was the end, truthfully. You always imagine the worst.” (Caregiver 1)
	“In March they call me and tell me that I am on hold. That I do not meet the requirements for the posttransplant phase. Many do not know that after the transplant the foundation no longer pays for your insurance and medications.” (Patient 2)
Lack of communication regarding timeline	“I lost hope many times. Like I said, I got depressed, I didn’t want to do anything. Sometimes I didn’t even eat. I didn’t think I was going to get the transplant.” (Patient 4)
	“They put me on a treadmill right? They didn’t give me much of an explanation…Yes, I’m telling you, there was not much information about it.” (Patient 11)
	“We started protocol in January. And there were these appointments where they gave them to you up until June.…It is so much time.” (Caregiver 2)
Concern for family limiting living donation	“If I die, I’m already old, nobody needs me, but they have children that need them, and I didn’t want to risk either of them.” (Patient 6)
	“Nobody wants to hurt the family…so I didn’t want it to be my wife because I didn’t want to affect her.” (Patient 11)
	“They said if [my daughter] gets married, it would take time and they wouldn’t be able to [donate]. We never spoke to the team.…This was our decision, it’s better not to.” (Caregiver 3)
	“I didn’t want them to [donate] because if one day the kidney fails them, they’ll end up like me. And I wouldn’t want my kids to feel what I felt.” (Patient 14)
Posttransplant improvements	
Ability to work after transplant is critically important given immigration status	“I can work better. I feel better for work, I work longer hours and all that. I feel much better for everything.” (Patient 3)
	“I said to myself, ‘Now that I have my transplant, maybe I can reactivate myself in society. To be productive. To be part of this society and feel useful.’” (Patient 1)
	“Having my transplant, I can now work and maintain myself.” (Patient 6)
Autonomy with transplant improves mental health	“Everything changes a lot. It changes your life. Transplantation is to live again, to be born again. For me, what happened in my life after the transplant is something magical.” (Patient 2)
	“It has been a good experience because the main thing is that he no longer receives dialysis. So the patient is now 100% in charge of his health.” (Caregiver 5)
	“Life is easier, with more expectations of moving forward, of enjoying life, right? When you are on dialysis it is like everything pauses.” (Caregiver 2)
	“Now I have a better chance to do everything, to take care of myself, to take care of the family and to take care of her more than anything else. We changed a lot.” (Caregiver 6)
Vigilance in maintaining transplant	“We have to take care of ourselves to protect it, but you can now indulge in food and know that it will not affect you quickly.…You no longer have the pressure that if you don’t go to dialysis, you could be between life and death.” (Patient 1)
	“Now we have to take precautions. So it’s not that now that you have a transplant and you can drink soda or you can now do whatever you want. No, because now you have to take care of your kidney.” (Caregiver 2)
	“I had to take care of myself just like I was taking [care] of myself before.” (Patient 12)
Transplant facilitators	
Self-advocacy	“I was always on top of the doctors. Maybe they didn’t like me. But I said, ‘It’s my life. It’s my health. And I want to live and I’m going to fight for that transplant.’” (Patient 1)
	“I finished the protocol very quickly because I was making one appointment after other.…If we want the transplant, we have to be like, ‘Hey, I’ve already had this test. What’s next? What am I missing?’” (Patient 2)
	“I don’t like to be left with any doubts, I am very inquisitive…I had any concerns or if my husband had any discomfort, I could write it all down, and they would answer all our questions.” (Caregiver 5)
Spirituality and optimism	“Be positive. I say that situations are unavoidable but my attitude is optional. My attitude doesn’t make me feel better. On the contrary, it knocks me down. So make an effort and don’t lose your dreams because, to those who believe, everything is possible.” (Patient 11)
	“I only think about today and tomorrow. Obviously you want the best for the future, but it’s about living for today.” (Caregiver 5)
	“I started to learn about the disease and I didn’t see it as a disease but as a different lifestyle. I could lead a normal life as long as I saw it that way.” (Patient 2)
	“I’ve always seen and I know that she is very strong. It’s part of her character. It’s strong. And she is, I can say she is determined with what she is going to do.” (Caregiver 1)
Peer support	“We met a person who had already been transplanted. And I asked her questions, right?…And she gave us all the information.” (Caregiver 2)
	“I was motivated to help all the people who were there to look for information. I said, ‘These people need the support, they need to find it. I’m going to find it. I’m going to understand the disease and look for the consequences.’…If we can fight for them, why not do it?” (Patient 1)
	“Try to understand each other because this is not easy at all and it’s nothing more than motivating them to keep going, to try their best.” (Caregiver 3)
	“Now we are more aware that we can help people who are in Mexico and people who are here, regardless of the situation of each person.…It used to be a very closed thing, only the family members and the people who were living that situation were the ones who really knew what they were going through. They didn’t talk about it as much.” (Caregiver 7)

^a^ Parentheses and number following each quote refers to the participant interview number. Caregiver numbers do not match the patient numbers.

### Limited Kidney Replacement Therapy Education While Receiving Emergency Hemodialysis

#### Lack of Awareness of Kidney Disease and Treatment Options

Participants learned about their kidney failure diagnosis after developing symptoms requiring dialysis. Many reported limited education regarding kidney failure and kidney replacement therapy. For example, many did not know that kidney failure was a permanent process. One patient stated, “I did not know how difficult it was going to be and that it was a process that was going to last for the rest of my life.” Participants reported no education about transplant while receiving emergency dialysis. One caregiver recalled, “They didn’t talk to us about transplants or anything, they just told us that they couldn’t take care of her there.”

#### Discriminatory Kidney Replacement Therapy Education Due to Immigration Status

Participants perceived their care received in the hospital was discriminatory because of their immigration status. Participants were directed to go back to their home country to receive care, and when they had questions about how they could receive insurance or dialysis care, they received little help. Transplant was rarely discussed, but when it was, many were told they would never have the option. One caregiver stated, “The only thing they told us was, there is nothing here for you. Why don’t you go back to your country? Here you will never be able to have a transplant.”

### Hope for Transplant Once Receiving Scheduled Dialysis

#### Prospect of Transplant Through Scheduled Dialysis 

Improvement in symptoms and schedule with thrice-weekly dialysis allowed patient participants to resume a more normal life, allowing them to consider the prospect of receiving a transplant. Participants described how a regular schedule allowed for a more normal life and more time with family and mitigated anxiety regarding the previous unpredictable dialysis schedule. Caregivers described that this change provided hope that a transplant might be possible.

#### Family and Quality of Life as Transplant Motivators

Motivators for transplant included understanding that a transplant would improve their quality of life, their ability to work, their sense of feeling useful and autonomous, and being with family. One participant reflected, “I saw it as a possibility to improve my life, to be able to work.”

### Transplant Education and Health Insurance After Transition to Scheduled Dialysis

#### Inadequate Transplant Education in Dialysis Clinic

In the dialysis center, participants received education that transplant was an option to treat kidney failure (which was often not discussed during emergency dialysis) but were often told this would be difficult or near impossible without insurance and did not receive information on how to procure insurance. Others reported receiving misinformation regarding the requirements for transplant. One patient recalled, “They said I needed to have $100,000.…And then she said I needed legal documents.” Many recalled thinking that transplant would be impossible for them as a result.

#### Peer-to-Peer Transplant Education

Participants reported that a main source of transplant education was learning from peers about the opportunities for transplant. Hearing stories from peers who had successfully navigated the process allowed patients to gain motivation to pursue transplant themselves.

#### Peer-to-Peer Communication Regarding Availability of Private Health Insurance

Participants reported that their dialysis peers informed them about the availability of a charity that funded private health insurance. Around this time, a peer support group for individuals of undocumented immigration status receiving dialysis started information sessions regarding insurance options. One participant described the process: “Information about the transplant, that happened like after 5 or 6 years, that was when they recommended an insurance option for us who don’t have a social security number.…But at first, nothing, I think that was because we didn’t have a chance to get a transplant with no insurance.”

### Uncertainty During Transplant Evaluation

#### Difficulty Navigating the Evaluation and Wait-Listing Process

Participants reflected that the evaluation process was an anxious time after persevering with emergency dialysis. Participants described fear of being ineligible due to poor test results as a feeling similar to the anxiety they felt trying to meet laboratory requirements to receive emergency dialysis. Caregivers described anxiety when there were complications with testing as reminiscent of the uncertainty they felt for the patient in the emergency dialysis days. Another major concern was losing insurance and no longer being eligible for transplant. One caregiver stated, “I worried that they were going to take [the insurance] away from him…they could also say no again and he would have to go back to [emergency dialysis].”

#### Lack of Communication Regarding Timeline

Participants described poor communication regarding the evaluation and wait-listing process, especially regarding the long timeline for testing and the waiting list. After years undergoing dialysis, many thought the path to transplant would be fast after procuring insurance, and many felt anxious waiting to find out whether they were eligible and not understanding why the tests were spaced so far apart or why they were being performed. One participant described, “There was a lack of communication.…You’re left with uncertainty because you don’t know what’s next.”

#### Concern for Family Limiting Living Donation

Patient participants described multiple barriers to living donation. Many were afraid of a bad outcome for their potential donors and were concerned about the burden they would be on their family.

### Posttransplant Improvements

#### Ability to Work After Transplant Is Critically Important Given Immigration Status

Participants described being able to work more after their transplant; they had more time without the burden of dialysis, and their physical symptoms were improved. Participants described that this improved ability to work was important to their self-worth because they have limited safeguards and migrate to the US to work and send money home. As one patient said, “I consider it a miracle of God that gave me a second chance to reintegrate into society and be useful.”

#### Autonomy With Transplant Improves Mental Health

After transplant, participants described feeling a sense of control over their future in contrast to living from week to week when receiving emergency dialysis. Many caregivers had described a sense of helplessness and lack of autonomy when their loved one received emergency dialysis and a similar sense of anxiety throughout the transplant process. After transplant, one caregiver noted, “My day to day life is mine now.”

#### Vigilance in Maintaining Transplant

Participants described being careful about their health and medications. This vigilance was due to fear of having to return to dialysis and years of experience needing to be extremely careful about diet due to only receiving dialysis once a week.

### Transplant Facilitators

#### Self-Advocacy

Patients highlighted the value of self-determination as a key factor in ultimately receiving a transplant, especially in the education stage. Participants’ involvement included doing their own internet search on transplant and asking lots of questions regarding insurance and testing because these issues were not always explained.

#### Spirituality and Optimism

Participants described using strategies to maintain a positive outlook through the many barriers they faced en route to transplant. Strategies included living day to day, viewing their chronic disease as a lifestyle, using their religious faith, helping others, and gratitude. One participant reflected, “The first thing that helps you to keep your organ in good condition is the medication. The second thing is nutrition, and the third thing is your mentality.”

#### Peer Support

Participants reported that the support and navigation assistance from their peers were critical to the transplant process because they received limited education regarding transplant and insurance options from health care workers. Participants felt that seeing people like themselves facing the same challenges showed them it was possible. Many felt after transplant that they needed to become the source of this information for other people of undocumented immigration status receiving dialysis.

## Discussion

In this qualitative study, we found that individuals of undocumented immigration status received minimal formal transplant education or insurance navigation. Transitioning to scheduled dialysis facilitated a strong peer support network and improvements in health, which allowed participants to pursue transplantation through self-determination and easier access to important information. These findings imply that adequate transplant and insurance education and peer support may play a role in success with transplant. These findings may be used to implement improvements in access to support and education for individuals of undocumented immigration status with kidney failure, especially in areas where scheduled dialysis is not available.

The participants in our study received minimal predialysis kidney disease education and experienced discriminatory treatment while they relied on emergency dialysis, similar to prior studies.^15^ Patients had to take the initiative to educate themselves; however, the symptom burden of emergency hemodialysis led to a high caregiving burden on family and an inability to work.^6,7^ Combined with lack of appropriate education about transplant and insurance opportunities, this burden limited any ability to seek transplant information. Even after moving to scheduled dialysis, transplant and insurance education was minimal for our participants. In a study identifying barriers to transplant for individuals of undocumented immigration status in Illinois (where dialysis is provided statewide),^2^ kidney disease education and insurance education were not reported barriers.^16^ Our findings illustrate that in states without provisions for kidney transplant, formal education regarding transplant may be withheld from people of undocumented immigration status due to nonmedical reasons. Furthermore, many participants were told to go back to their country to receive dialysis, although many countries in Latin America do not have uniform access to affordable dialysis.^17^ Regardless of their financial means, patients deserve the right to understand the options for their care.

A key area for education, information, and support for the undocumented community with kidney failure in our study was their community. Indeed, most participants first heard about options for private health insurance through a community peer support group.^18,19^ Peer support groups for patients with kidney disease have demonstrated improvements in self-efficacy, emotional well-being, decision-making, and development of coping strategies^19,20,21^; in a peer group for individuals of undocumented immigration status receiving emergency dialysis, the peer support allowed patients support to improve care and resilience, including self-advocacy, self-motivation and optimism, and kidney disease education.^17^ The participants in our study reported that self-efficacy was a key component in their pursuit of transplant, a skill that may have been augmented by seeing others like themselves succeed with transplant. Furthermore, transplant recipients described stepping into a peer support role for other individuals of undocumented immigration status after their transplant.

The key motivation for our participants in seeking transplant was improvement in quality of life, which included a sense of purpose and returning to work. Although this is a common motivator for all patients receiving dialysis, people of undocumented immigration status are often young and working age and are motivated to work for multiple reasons, including cultural expectation of providing for the family and lack of disability benefits for dialysis that are available to US citizens. These issues are important to acknowledge given a key ethical argument against providing transplant for individuals of undocumented immigration status is that they have no fiscal right to health care benefits because they do not pay into the system, despite evidence showing these individuals do pay taxes,^22^ which are largely used to subsidize US citizens’ health care.^23^ In addition, inclusion of scheduled dialysis and transplant for people of undocumented immigration status with kidney failure is fiscally sensible.^24,25^ Transplant recipients of undocumented immigration status have similar or better transplant outcomes compared with citizen recipients,^26,27,28^ contribute to the organ pool,^29^ and often report having at least 1 living donor candidate.^25^ Our study demonstrates that transplant recipients of undocumented immigration status are motivated to contribute to society^25^ through working and are better able to work then when they were receiving dialysis.

### Limitations

Our study has limitations. First, these results may not be transferable given the unique experience of receiving both emergency dialysis and scheduled dialysis. Second, our participants were able to pass the financial screen for transplant and may not reflect the experiences of those with greater socioeconomic challenges. To pass the financial screen, the patient needs to demonstrate the ability to finance insurance copayments after the first calendar year of transplant, when their charity-funded insurance ends.^3,4^ Third, our participants did not report issues with language and culture concordance during the transplant process due to the advent of a culture-concordant transplant center in Colorado,^30^ which may not be transferable to other centers.

## Conclusions

In this qualitative study of transplant recipients of undocumented immigration status and their caregivers, we found that transplant recipients who previously received emergency dialysis received little to no formal kidney or transplant education and their peer community was a major source of education, motivation, and support en route to transplant. Improving education on transplant regardless of systemic barriers may be an important next step toward patient-centered kidney care for this population.

## Notes

Supplement 1. **eTable. **Interview Guide for Transplant Recipients and Caregivers **eFigure.** Thematic Schema Illustrating Associations Between Transplant Facilitators and Navigation of Transplant Process for Undocumented Immigrants Receiving Emergency Dialysis

Supplement 2.
Data Sharing Statement
