# Supplementary material for: The Transplant Experience for Undocumented Immigrant Patients Formerly Receiving Emergency Dialysis and Caregivers
Source: JAMA Netw Open. 2024 Feb 29;7(2):e2354602. doi: 10.1001/jamanetworkopen.2023.54602 (PMC10905299; doi:10.1001/jamanetworkopen.2023.54602)
Supplement: Supplement 1. — eTable. Interview Guide for Transplant Recipients and Caregivers eFigure. Thematic Schema Illustrating Associations Between Transplant Facilitators and Navigation of Transplant Process for Undocumented Immigrants Receiving Emergency Dialysis [file jamanetwopen-e2354602-s001.pdf]

Supplementary Online Content

Rizzolo K, Rockey N, Camacho C, Gardner C, Giusti S, Cervantes L. Transplant experience for undocumented immigrant patients and caregivers formerly receiving emergency dialysis. *JAMA Netw Open*. 2024;7(3):e2354602. doi:10.1001/jamanetworkopen.2023.54602

eTable. Interview Guide for Transplant Recipients and Caregivers

eFigure. Thematic Schema Illustrating Associations Between Transplant Facilitators and Navigation of Transplant Process for Undocumented Immigrants Receiving Emergency Dialysis

This supplementary material has been provided by the authors to give readers additional information about their work.

© 2024 Rizzolo K et al. *JAMA Network Open.*

eTable. Interview Guide for Transplant Recipients and Caregivers

Interview guide for transplant recipients

Pre-transplant

1. *Explore the dialysis experience.* (Prompts: Can you describe your dialysis experience prior to receiving a transplant?)
2. *Explore effects of dialysis on family/work.* (Prompts: Can you describe how kidney disease impacted your ability to work? How did your kidney disease impact your family?)
3. *Explore transplant education.* (Prompts: Did you receive information about the transplant process? When did you receive this information? Did you receive any information about living donation?)

Transplant

4. *Explore motivations for transplant.* (Prompts: What are the main reasons you chose to undergo transplant? What were your biggest hopes and fears or doubts regarding transplant?)
5. *Explore challenges of the transplant evaluation process.* (Prompts: Can you share good and bad experiences when you were going through the evaluation and testing process? What were the main challenges you faced in going through the transplant evaluation process?)
6. *Explore social barriers, such as financial/insurance, language, family.* (Prompts: Did you experience any challenges with transplant evaluation? Did you have any issues or concerns with testing? How was your family impacted by going through the transplant process?)
7. *Explore the peri-transplant period.* (Prompts: How much time was there between your decision to go on the transplant list and when you finally received your transplant? Were there any delays? What was the hospitalization and recovery after surgery like?)

Post Transplant

8. *Explore post-transplant life.*
    a. *Discuss life changes after transplant.* (Prompts: How has your life changed after receiving the kidney transplant, such as work, symptoms, medical care?)
    b. *Explore quality of life.* (Prompts: How has your mood and level of stress been affected? Have you noticed an effect on your family/ those in your social circle?
    c. Is there anything you didn't expect?
9. *Is there anything you would share with a person on dialysis considering a transplant?*
10. *From your perspective, how could we help more patients begin the transplant process?*

© 2024 Rizzolo K et al. *JAMA Network Open.*

Interview guide for caregivers of transplant recipients

Pre-Transplant:

1. *Explore the caregiver dialysis experience.* (Prompts: What did a regular day look like for you and your loved one prior to transplant? What did providing care at home look like?)
2. *Explore the effects of dialysis on the caregiver.* (Prompts: What did a good day look like prior to the transplant? What did a bad day look like prior to the transplant? What were your biggest concerns or fears prior to the transplant?)

Transplant:

3. *Discuss the transplant education and evaluation process.* (Prompts: How did you learn about the opportunity for transplantation? Was there anything that was difficult to understand about the transplant evaluation? What were your biggest concerns or fears during the transplant process?)
4. *Discuss the peri-transplant period* (Prompts: What was your role during the hospitalization and post-op for the surgery?)

Post-Transplant:

5. *Explore quality of life post-transplant.* (Prompts: What does a good day look like for you and your family? What does a bad day look like for you and your family?)
6. *What advice would you give to caregivers of people on dialysis considering a transplant?*

© 2024 Rizzolo K et al. *JAMA Network Open.*

Supplemental Figure. Thematic schema illustrating associations between transplant facilitators and navigation of transplant process for undocumented immigrants receiving emergency dialysis.

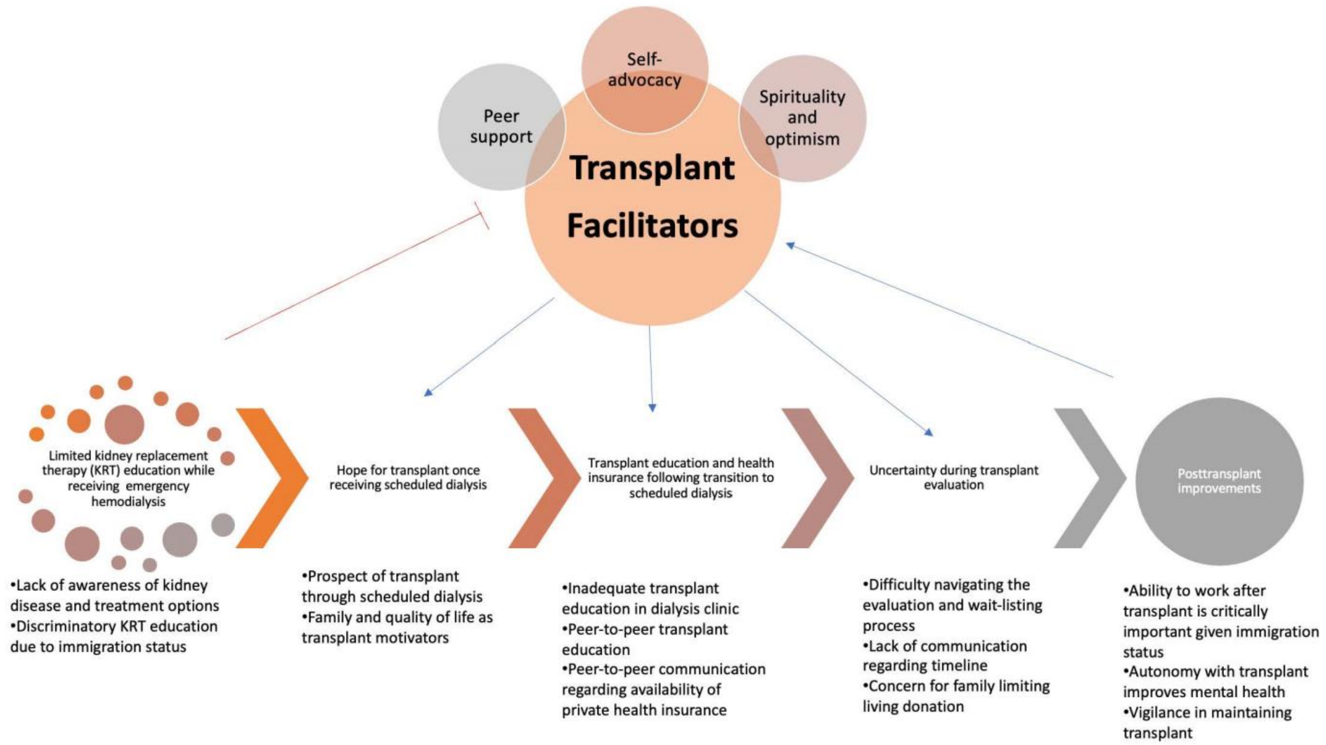

© 2024 Rizzolo K et al. *JAMA Network Open.*
