# Supplementary material for: The Transplant Experience for Undocumented Immigrant Patients Formerly Receiving Emergency Dialysis and Caregivers
Source: JAMA Netw Open. 2024 Feb 29;7(2):e2354602. doi: 10.1001/jamanetworkopen.2023.54602 (PMC10905299; doi:10.1001/jamanetworkopen.2023.54602)
Supplement: Supplement 2. — Data Sharing Statement [file jamanetwopen-e2354602-s002.pdf]

Data Sharing Statement

Rizzolo. The Transplant Experience for Undocumented Immigrant Patients Formerly Receiving Emergency Dialysis and Caregivers. *JAMA Netw Open*. Published March 01, 2024. doi:10.1001/jamanetworkopen.2023.54602

Data
Data available: Yes
Data types: Deidentified participant data
How to access data: available on request from the corresponding author.
When available: With publication

Supporting Documents
Document types: Informed consent form
How to access documents: available on request from the corresponding author.
When available: With publication

Additional Information
Who can access the data: available upon request
Types of analyses: for research purposes
Mechanisms of data availability: with investigator support
